# Comparison of reproducibility and workability of single and adjacent implant placement protocol under dynamic real time navigation systems between operators: A clinical trial

**DOI:** 10.1016/j.jobcr.2025.08.003

**Published:** 2025-08-09

**Authors:** Vamshi Nizampuram, Sahana Selvaganesh, Thiyaneswaran Nesappan

**Affiliations:** a Department of Implantology, Saveetha Dental College and Hospitals, Saveetha Institute of Medical and Technical Science, India; b Saveetha Dental College and Hospitals, Saveetha Institute of Medical and Technical Science, Saveetha University, Chennai, India

**Keywords:** Real-time navigation, Dynamic navigation, Guided implant surgery, Implant placement accuracy

## Abstract

**Introduction:**

Dynamic navigation (DN), a computer-assisted technique integrating CBCT data and real-time video, has emerged as a promising approach to place implants in the recent years. This study aims to evaluate the consistency and ease of use of a dynamic navigation system for implant placement by comparing the accuracy in single and adjacent implant placements and workability achieved by three different operators.

**Methods:**

This study included Forty-eight patients requiring dental implants, total of sixty implants were randomly assigned to 3 operators of varying experiences, the implants were planned and placed under DN. The accuracy of implant placement were measured in terms of mesio-distal, apico-coronal displacement and angulations using Evalunav application (*Navident, Claronav, Canada*). Secondary outcome variables are the number of errors encountered during the procedure and the time taken for the procedure by different practitioners. Kruskal Wallis Test followed by the Post hoc Mann Whitney *U* test. The level of significance was set at P < 0.05.

**Results:**

There were no significant differences in the accuracy of single implants (P > 0.05). For adjacent implants (T1), the displacement in mesiodistal direction was significantly different (P = 0.003) and also for apico-coronal position of T1-abutment group when compared to controls with a P value of 0.026. Experienced surgeons had the highest error rates as well and longest time (18.27 ± 5.62 versus 15 min).

**Conclusions:**

The operating surgeon do not determine the accuracy rather the navigation system comes with a steep learning curve that needs to be acquired prior to practicing the same.

## 1 Introduction

Dental implants have emerged as a popular and successful procedure and technique for restoring lost teeth. However, a great level of accuracy and precision is required for successful implant placement.^1^ Dynamic navigation (DN) has become a potential approach for enhancing the precision and predictability of dental implant placement in recent years.^2^ The effectiveness and repeatability of the methodology for implant placement under dynamic navigation require to be explained in detail with enhanced evidence and improved sample size.

DN is a computer-assisted technique that merges the CBCT data and video recording in real-time, to accurately place an implant in a pre-planned position by using tracking devices and imaging technology to co-relate the data fed into the software and the jaw that the camera is tracking. The tracking devices are utilized to direct the surgeon in real-time during the procedure, ensuring that the implant is positioned exactly as intended^3^ in the virtual model.

DN offers high accuracy and precision, with studies showing it can increase implant insertion accuracy by up to 45 % compared to conventional freehand approaches.^4^ It can help surgeons navigate complex anatomical structures with precision and ease in situations when the patient has complex anatomical structures in the planned site or restricted vision.^5^^,^^6^

Numerous research studies have examined the accuracy of dynamic navigation during implant placement. These experiments have shown that dynamic navigation offers a high degree of repeatability, i.e., the method may be consistently used across many scenarios with little variation between planned and actual implant locations. According to Battista et al. dynamic navigation is an accurate and consistent technique for implant placement.^7^ This study aims to compare the workability and repeatability between two strata of operators under Dynamic navigation.

Dynamic navigation is a useful tool for implant placement due to its sequence of operation and seamless incorporation into a dental practice's clinical workflow. While initial training may be necessary, the majority of doctors can easily master it. It provides advantages in terms of accuracy and precision in implant placement, although some initial setup and calibration are necessary. Overall, dynamic navigation workflow is simple and can be quickly incorporated into clinical practice. Another study by Jorba-García et al., 2019, revealed that dynamic navigation was simple to learn and use, even for less experienced doctors. Even in the hands of inexperienced users, dynamic navigation offered a high degree of accuracy and precision, according to the study.^8^

This study aimed to compare and evaluate whether three different operators could reliably achieve accurate and easy-to-use implant placement using a dynamic navigation (DN) system. Accuracy measured in terms of displacements and angular deviations to the planned implant positions and the ease of use in terms of the time taken to set up and operate plus the errors that occurred during the surgical procedure. The null hypothesis was that there would be no reproducibility of the accuracies and ease of workability of the DN implant placement protocol. The alternative hypothesis was that there would be reproducibility of the accuracies and ease of workability of the DN implant placement protocol.

## 2 Materials and methods

### 2.1 Study design

#### 2.1.1 Study design and ethical approval

This was a single-center, randomized clinical trial conducted at a private university set up from October 2022 to February 2023. The study was approved by the Institutional Ethical Committee (reference number - PhD/IMP/1904/22/017). The research adhered to the ethical principles governing human experimentation, aligning with the Helsinki Declaration of 1975, as revised in 2000. CONSORT guidelines to report the trials was adhered.

#### 2.1.2 Study participants

**Fig. 1:**
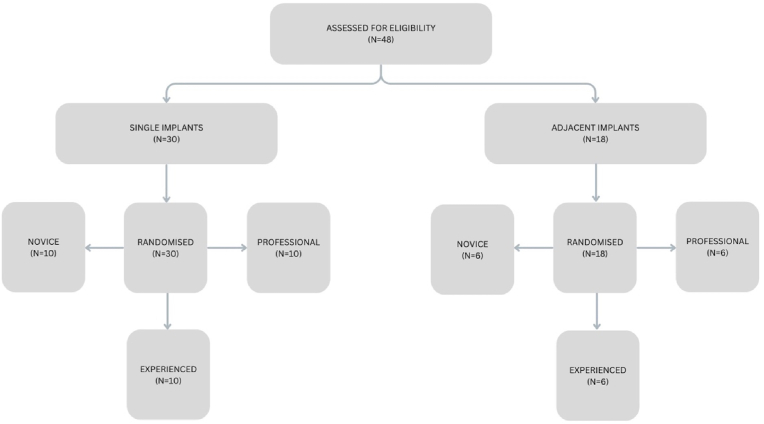
Flowchart showing the study design, and the distribution of the patients into groups.

Patients requiring a single or adjacent teeth replacement were included in the study (Fig. 1). Patients requiring full mouth implant replacements were excluded. Written informed consent was obtained from all participants before enrollment. Patients were randomized to the study and control groups before the start of the procedure using a computerized randomization scheme using *Random Allocation Software, Microsoft, Redmond, Washington, United States*.

#### 2.1.3 Sample size calculation

The sample size was calculated to be 48 patients using G∗Power version 3.1.5, based on an apical deviation of the implants with a confidence level of 90 %, a Z-score of 1.65, an effect size of 6.7, and an α error of 0.01.

#### 2.1.4 Randomization and blinding

Three operators with varying experience in placing implants participated in the study (Table 1):

Operator 1: Beginners, with six months of experience.

Operator 2: Practicing implantologist, with seven years of experience.

Operator 3: Experienced surgeon, with twenty two years of experience.

Each operator worked on sixteen patients, 6 of whom required adjacent implants and 10 of whom required a single implant procedure. The accuracy of the implant placement was assessed by an independent assessor who was blinded to the surgical approach.

### 2.2 Execution of the study

#### 2.2.1 Pre-operative planning protocol

Following the preliminary screening, a Cone beam computed tomography (CBCT) scan (CARESTREAM CS 9600, Carestream Dental, Rochester, NY, USA) was procured and an intraoral scan (RUNYES IOS 11, Runyes Technology Co., Ltd., Shenzhen, China) were performed as part of the diagnostic procedure. DICOM (Digital Imaging and Communications in Medicine) and STL data (Standard Tessellation Language) Were retrieved. The dynamic navigation software Navident version 3, was used to plan the implants. For each case, the implants of optimum length, width, and angulation were selected from the Navident planning software. On the day of the surgery, the following procedures were followed.

**Fig. 2:**
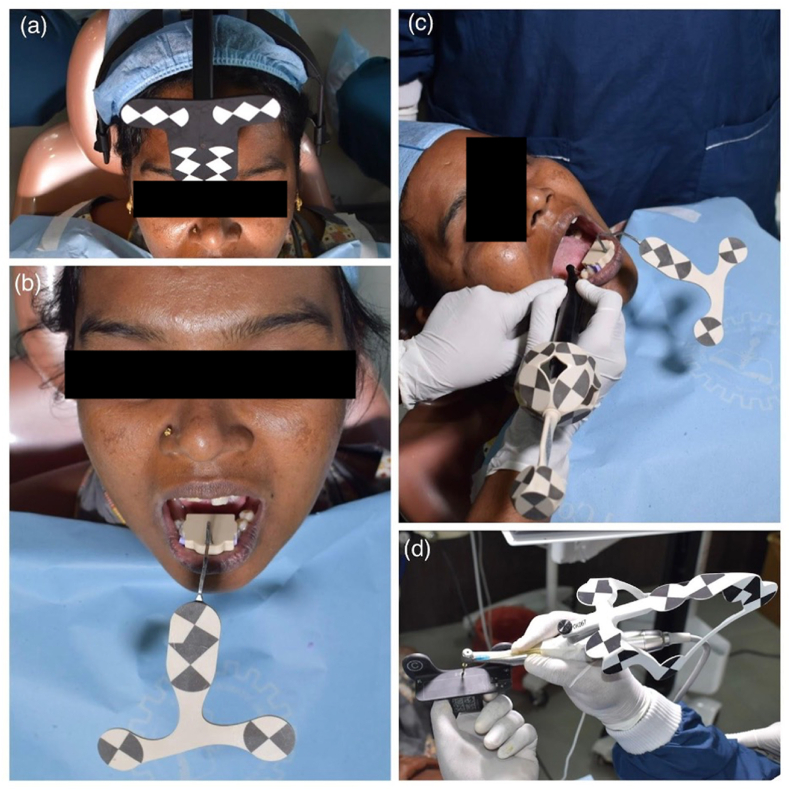
2A) Jaw tracker for the mandibular Arch, 2B) Head Tracker for the Maxilla 2C) Tracer Tooth Calibration, 2D) Drill tip Calibration.

On the day of surgery, strict sterilization protocols were observed. The patient was comfortably seated, and the Dynamic navigation unit was prepared. Depending on the arch, either a jaw tracker was secured with bite registration paste (for the lower arch, or an "I" tracker for mandibular anteriors) or an upper jaw tracker was fitted over the head (for the maxillary arch, preferred for accuracy). The registration process involved two trackers: one on the patient's jaw and another on the handpiece, with their positions continuously monitored by stereo cameras to ensure real-time navigation. Once instruments were in place, jaw tracing was performed using a calibrated tracer tip. The drill tag on the handpiece was recognized via a barcode, and access calibration for the handpiece was completed, followed by sequential calibration of each drill (see Fig. 2). During drilling, the operator focused on the computer screen, using the real-time display to correct the drill's mesio-distal and bucco-lingual positioning and angulation to match the pre-planned implant site with an accuracy of within 1 mm and one degree. After sequential drilling, the implant itself was calibrated, allowing the Dynamic navigation unit to guide its precise placement into the osteotomy site. The surgical procedure was performed under lidocaine (1:80000), utilizing a flapless approach. Tissues at the implant site were cleared with a calibrated tissue punch, and decortication of bone was done with a precision drill, followed by sequential drilling after depth and width calibration. The implant was also calibrated before its final placement. Following the surgery, a post-operative CBCT was taken to assess the outcome and record the time taken for each procedural step, followed by operator feedback.

#### 2.2.2 Data collection

In this study, the predictor variables were the planned implant positions and the operators performing the procedure, with no control group for comparison. The primary outcome variables focused on the accuracy of implant placement, specifically measuring mesiodistal and buccolingual displacements, along with mesiodistal and apico-coronal angulations. These measurements were precisely determined using pre- and post-operative Cone Beam Computed Tomography (CBCT) scans, which were analyzed with specialized software to quantify the distances and angulations between the implants and surrounding anatomical structures. Secondary outcome variables included the ease of operation, assessed by recording the time taken for equipment setup and implant surgery using a stopwatch, and identifying errors encountered by the three operators, which were documented by a trained observer who noted the type, severity, and cause of each error.

#### 2.2.3 Single implants

**Fig. 3:**
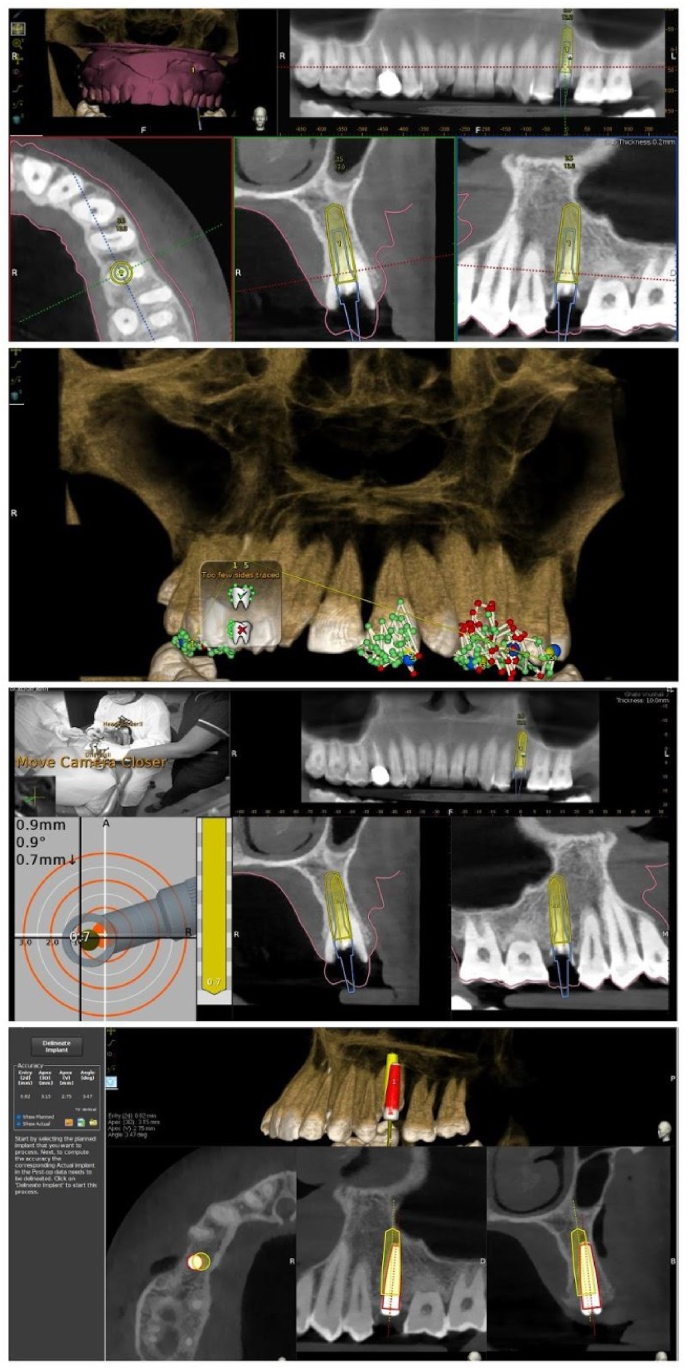
3A) Tracer Calibration, 3B) 3C) drilling sequence under dynamic navigation, 3D) Evalunav analysis (Planned and Implemented Implant Position of the First Implant).

The above-mentioned protocol was followed for single implant placement, and the following data were collected: DN unit setting up time, drilling time, implant placement accuracy, errors that occurred during the implant operation that required the Dynamic Navigation system to be recalibrated, and operator feedback was also collected after the procedure. An observer was assigned to use a stop clock to record the setup and drilling times. The observer was also entrusted with recording any issues that arose throughout the operation, which was verified using the video from the Navident software. A post-CBCT was carried out to evaluate implant placement accuracy, and the data was uploaded to the Evalunav software, which calculated implant placement accuracy in terms of Mesio-distal angulation, Mesio-distal displacement, Bucco-lingual displacement, and Apico-coronal displacement. The acquired data was then analyzed among the three operators, and the findings were interpreted (Fig. 3).

#### 2.2.4 Adjacent implants

The purpose of computing the data of adjacent implants was to see if there was any major variation when adjacent implants were placed, as well as to see if the setting up and operation times were extended when multiple implants were to be placed.

**Fig. 4:**
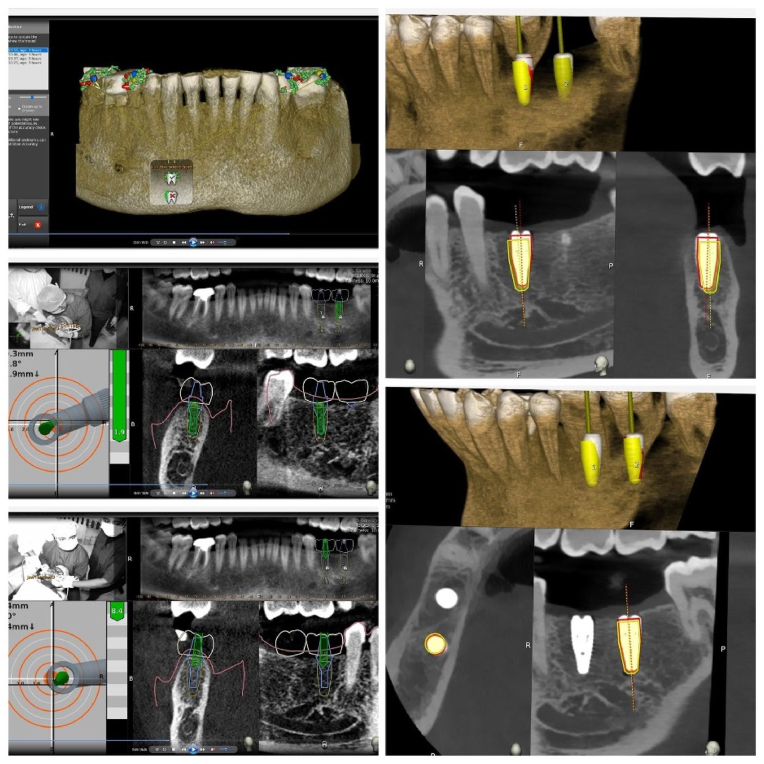
A) Tracer Anatomical calibration, 4B) first implant Placement, 4C) Second Implant Placement, 4D) Planned and Implemented Implant Position of the First Implant, 4E) Planned and Implemented Implant Position of the adjacent implant.

The methodology described above for the single implant was likewise followed for the adjacent implants, and the information collected was examined and interpreted (Fig. 4).

## 3 Statistical analysis

The data was analyzed using SPSS for Windows [*SPSS ver 26.0, IBM Corp., Armonk, NY*].

Continuous data between the groups for various clinical parameters was compared using the Kruskal Wallis Test followed by the Post hoc Mann Whitney *U* test. Data was presented using tables and graphs. The level of significance was set at P < 0.05.

## 4 Results

The descriptives such as the age of the patient included in the study and the gender of the patients are represented in (Table 1)., there is no association of these with the improvement or deceleration of the accuracy of the operators who were involved in the study.

**Table 1:** Comparison of the co-variates and predictor variable.

	Student (n = 16)	Intermediate Practitioner (n = 16)	Experienced Surgeon (n = 16)
Age	45 (3.33)	47 (4)	42 (5.25)
Gender	62.5 % Males, 37.5 % Females	56.25 % Males, 43.75 % Females	50 % Males, 50 % Females

n-number of samples.

### 4.1 Accuracy of single implant placed

Kruskal Wallis test showed non-significant data concerning the accuracy of placement of single implants among a student, practitioner, and an experienced surgeon (Table 2). The accuracy was measured based on Mesiodistal Angulation, Mesiodistal Displacement, Buccolingual Displacement, and Apico-coronal Displacement, and the Mean ± SD among the operators was also assessed.

**Table 2:** Comparison of mean of the clinical parameters between various operators for single implant placement.

	Student (n = 10)	IntermediatePractitioner (n = 10)	Experienced Surgeon (n = 10)	Kruskal Wallis H	P value
Mesiodistal Angulation	0.83 (0.67)	0.86 (0.74)	0.65 (0.56)	1.65	P = 0.43
					(NS)
Mesiodistal Displacement	1.63 (0.93)	1.60 (1.13)	1.78 (0.99)	0.389	P = 0.82
					(NS)
Buccolingual Displacement	0.80 (0.80)	0.65 (0.70)	1.03 (0.75)	2.18	P = 0.335
					(NS)
Apicocoronal Displacement	5.10 (3.53)	3.65 (3.16)	4.76 (2.79)	1.41	P = 0.49
					(NS)

n-number, NS-Not significant using Kruskal Wallis Test.

It was found that mesiodistal angulation and mesiodistal displacement of single implant was lowest for experienced surgeons while it was highest for students. However, this difference was not statistically significant (P = 0.43 and P = 0.82). In addition, buccolingual displacement and apico-coronal displacement were lowest among practitioners and highest for experienced surgeons. However, this was not found to be statistically significant (P = 0.335 and P = 0.49).

This implies that statistically there was no difference in values concerning various clinical parameters between students, practitioners, and experienced surgeons for single implants. In other words, the procedure was reproducible across different operators.

### 4.2 Accuracy of adjacent implants placed

#### 4.2.1 First implant (T1)

It was found that mesiodistal angulation and buccolingual displacement of T1 of the parallel implant were lowest among practitioners and highest for students. However, this difference was not statistically significant (P = 0.85 and P = 0.06). In addition, mesiodistal and apicocoronal displacement was highest among experienced surgeons and was statistically significant (P = 0.003 and P = 0.026).

A post hoc comparison between the operators for mesiodistal displacement revealed statistically significant differences between student and experienced surgeon (P = 0.004) and between practitioner & experienced surgeon (P = 0.004). This implies that the implant navigation system for T1 of parallel implants had similar results for students and practitioners. In other words, the procedure was not reproducible between all the operators (Table 3).

**Table 3:** Comparison of mean of the clinical parameters for Implant 1, of adjacent implants placed by different operators.

	Student (n = 10)	IntermediatePractitioner (n = 10)	Experienced Surgeon (n = 10)	Kruskal Wallis H	P value
Mesiodistal Angulation	0.98 (0.44)	0.97 (0.75)	1.19 (1.02)	0.326	P = 0.85
					(NS)
Mesiodistal Displacement	1.15 (0.33)	1.10 (0.43)	2.99 (1.18)	11.3	P = 0.003∗∗
Buccolingual Displacement	0.86 (0.68)	0.68 (0.61)	1.78 (0.94)	5.4	P = 0.067
					(NS)
Apicocoronal Displacement	1.57 (1.46)	4.20 (3.59)	6.39 (3.97)	7.31	P = 0.026∗

n-number; NS-Not significant, ∗P < 0.05, ∗∗P < 0.01 using Kruskal Wallis Test.

**Table 4:** Comparison of mean of the clinical parameters for Implant 2, of adjacent implants placed by different operators.

	Student (n = 6)	IntermediatePractitioner (n = 6)	Experienced Surgeon (n = 6)	Kruskal Wallis H	P value
Mesiodistal Angulation	0.79 (0.46)	1.48 (1.04)	0.69 (0.57)	2.16	P = 0.33
					(NS)
Mesiodistal Displacement	1.23 (0.89)	1.50 (0.88)	2.32 (1.23)	2.66	P = 0.26
					(NS)
Buccolingual Displacement	0.86 (0.68)	0.87 (0.84)	1.50 (0.90)	2.39	P = 0.30
					(NS)
Apicocoronal Displacement	2.66 (1.97)	6.33 (5.86)	6.44 (6.55)	2.47	P = 0.29
					(NS)

n-number; NS-Not significant, ∗P < 0.05, ∗∗P < 0.01 using Kruskal Wallis Test.

A post hoc comparison between the operators for apico-coronal displacement revealed statistically significant differences between student and experienced surgeon (P = 0.01) (Table 5). This implies that the implant navigation system for T1 of the parallel implant had similar results for students & practitioners and practitioners and experienced surgeons. In other words, the procedure was not reproducible between all the operators.

**Table 5:** Multiple comparisons between different operators for mesiodistal displacement and Apico-coronal displacement.

Clinical Parameters	Operators	P Value
Mesiodistal Displacement	Student and Practitioner	0.87
	Student and Experienced Surgeon	0.00∗∗
	Practitioner and Experienced Surgeon	0.00∗∗
Apicocoronal Displacement	Student and Practitioner	0.149
	Student and Experienced Surgeon	0.01∗
	Practitioner and Experienced Surgeon	0.15

Statistically significant at ∗P < 0.05 and ∗∗P < 0.01 using Mann Whitney *U* test.

#### 4.2.2 Adjacent implant (T2)

The results showed that there was no statistically significant difference in the median values of the parameters between the three groups. This suggests that the procedure was reproducible by all the groups (Table 4).

The specific parameters that were measured were mesiodistal angulation (P = 0.33), mesiodistal displacement (P = 0.26), buccolingual displacement (P = 0.3), and apicocoronal displacement (P = 0.29). For all four parameters, the students had the lowest mean values, followed by the practitioners, and then the experienced surgeons. However, the differences between the mean values for the three groups were not statistically significant.

These results suggest that the navigation method is a reproducible procedure that can be used by surgeons of all levels of experience. The method is accurate and can be used to place parallel implants with a high degree of precision.

#### 4.2.3 Time taken to calibrate and drill

To compare the times taken to set up and the time taken to drill, an ANOVA, Tukey's posthoc test was done, which showed that the experienced surgeon took the most time to set up and drill (18.27 ± 5.62 min), compared to the student (11.53 ± 2.88 min) and the practitioner (10.8 ± 2.68 min), who on the other hand got used to the software and were at comparable timings.

#### 4.2.4 Errors during and after the procedure

An observer was appointed to monitor and record any mistakes that occurred throughout the implant procedure. 26.6 % of the cases treated by the student had a camera positioning error, followed by 6.65 % for the jaw tracker fixing error and 6.65 % for the drill tag not being detected. Intermediate practitioners experienced camera positioning error in 26.6 % of cases, 13.3 % error in jaw tracker fixing and re-tracing and in 6.65 % of cases the drill tag was not detected by the camera. The experienced surgeon had 33.3 % of camera positioning errors, 20 % of the patients had a problem with the jaw tracker that was fixed, and 5.2 % of the patients had a problem with the drill tag not being detected.

## 5 Discussion

Single implant placement achieved similar accuracy levels between students and practitioners and experienced surgeons when using DN systems. Previous findings by Battista et al. proved that navigation systems provide better placement accuracy than traditional freehand procedures.^7^

Results showed that experienced surgeons achieved different values than students and practitioners when placing adjacent implants in both mesiodistal (P = 0.003) and apico-coronal (P = 0.026) directions. Placing adjacent implants presents several difficulties according to these findings because operators need better anatomical knowledge and precise control. Vetern surgeons demonstrated higher placement deviation probably because they struggled with adjusting to new technology procedures despite having mastered traditional approaches. The study results are comparable with Jorba-García et al.‘s research (Entry 2D (p = 0.014), apex 3D (p = 0.008) and angulation deviation (p < 0.001)) that demonstrates dynamic navigation achieves accurate outcomes regardless of experiential surgeon level.^8^

Experienced surgeons spent longer performing setup operations and drilling by 18.27 ± 5.62 min compared to 11.53 ± 2.88 min used by students and the 10.8 ± 2.68 min used by practitioners. The difference in operational time indicates that experts who rely on traditional methods experience bigger challenges with learning DN technology. The findings of Sun et al. showed that device navigation decreases placement time yet students and practitioners who establish routines experience an initial delay in their procedure duration.^9^ The inclusion of experienced surgeons were necessary to assess the steep learning curve associated with Dynamic Navigation^10^

The most common procedural error among all practitioner groups appeared to be camera position problems which experienced surgeons reported most frequently at a rate of 33.3 %. Research indicates that experienced surgeons frequently miss important DN system technical elements because they trust their surgical competence too strongly according to Pellegrino et al. (p < 0.05).^11^^,^^12^ as they explain that navigation system adaptation demands time regardless of surgical background.

DN serves its highest value in complex anatomical locations where critical elements including the inferior alveolar nerve need to be avoided. A 2-mm safety margin to protect vital areas should be established since differentiations larger than 1 mm were detected. The situation emphasizes why computer-directed implant surgery is crucial because it provides superior accuracy as well as lower procedure-related complications during complex procedures.^13^, ^14^, ^15^

The study showed that DN systems present notable benefits alongside some restrictions. The usage of DN systems involves longer surgical procedures together with calibration steps and operator learning challenges as well as discomfort to patients from tracking devices and registration mismatches. DN provides greater benefits than its limitations make it suitable for use in complex implant procedures.

The establishment of DN systems enables both novice and experienced surgeons to achieve precise implant placement but surgeons need extra training before setting adjacent implants. The intraoperative adaptation possibilities of this system together with minimal access procedures help both patients and staff experience lower postoperative pain and shorter healing periods.

The future of dynamic navigation (DN) in implantology hinges on two key areas, improved training and technological advancements. This study revealed a steep learning curve for experienced surgeons, particularly in complex adjacent implant cases, suggesting a need for standardized education and training protocols to help operators of all levels master the system. Future research should focus on developing structured training modules and certifications to ensure proficiency. In addition, further long-term studies are needed to assess the durability and patient outcomes of DN-guided implants. Technologically, the high rate of procedural errors highlights the need for more robust tracking systems, intuitive software, and potentially augmented reality features to enhance real-time guidance. By refining both the training and the technology, the goal is to make the system reliable that the operator's prior experience becomes irrelevant, solidifying DN's role as a universal and highly precise tool in dental implant surgery.

The research study included various limitations that require recognition. The study has a significant limitation since it lacked a group that received standard implant placement using traditional methods which hinders sufficient comparison between DN and traditional implantology. The statistical power could have been impacted due to the small number of operators in each group. All operators participated with the DN system in their first experience thus their performance could have been affected by their inexperience with the system even though they already mastered implant placements. The interpretation of findings was limited by the evaluation of only a single DN system (Navident) since other platforms could produce dissimilar results.

The results show single implant placement using DN systems produces accurate results that operators with varying experience levels can achieve and adjacent implant placement remains challenging for surgeons with new technology experience. All users of the DN system need to complete a learning curve regardless of their prior experience in implant placement. Long-term performance evaluation of DN-guided implants requires further research along with testing various navigation systems and creating standardized education methods to maximize their application in dental practice.^16^

## 6 Conclusion

This research demonstrates that the Dynamic Navigation (DN) system facilitates consistent accuracy in single implant placement across operators of varying experience levels, indicating that the procedure's reproducibility for single implants is not significantly affected by the surgeon's prior experience. However, for adjacent implant placement, particularly for the first implant (T1), significant differences in mesiodistal and apico-coronal displacement were observed, predominantly with experienced surgeons showing higher deviations. This suggests that while DN offers substantial benefits, it presents a notable learning curve, especially for complex procedures like adjacent implant placement, where experienced surgeons, accustomed to traditional methods, may take longer to adapt to the new technology and consequently exhibit higher error rates and longer operational times. Ultimately, the study concludes that while DN systems offer enhanced accuracy and are a valuable tool in implantology, proficiency in their use requires dedicated training and adaptation, irrespective of a surgeon's pre-existing experience. Further research with diverse navigation systems and standardized training protocols will be crucial to maximize the widespread applicability and long-term success of DN-guided implant surgery.

## Ethical approval

The study was approved by the Institutional Ethical Committee (reference number PhD/IMP/1904/22/017). The research adhered to the ethical principles governing human experimentation, aligning with the Helsinki Declaration of 1975, as revised in 2000.

## Sponsorship

This research did not receive any specific grant from funding agencies in the public, commercial, or not-for-profit sector's.

## Funding

This is not a funded study.

## Declaration of competing interest

The authors declare that they have no known competing financial interests or personal relationships that could have appeared to influence the work reported in this paper.

## References

[1] Hama Dler Raouf, Jaza Mahmood Bayad (2023). Comparison of accuracy between free-hand and surgical guide implant placement among experienced and non-experienced dental implant practitioners: an study. J Periodontal Implant Sci.

[2] Block Michael S., Emery Robert W., Lank Kathryn, Ryan James (2017). Implant placement accuracy using dynamic navigation. Int J Oral Maxillofac Implants.

[3] Stefanelli Luigi V., Mandelaris George A., Franchina Alessio (2020). Accuracy of dynamic navigation system workflow for implant supported full arch prosthesis: a case series. Int J Environ Res Publ Health.

[4] Pellegrino Gerardo, Taraschi Valerio, Andrea Zacchino, Ferri Agnese, Marchetti Claudio (2019). Dynamic navigation: a prospective clinical trial to evaluate the accuracy of implant placement. Int J Comput Dent.

[5] Rueda González, Ramón Juan, Galparsoro Catalán Agustín (2023). Accuracy of computer-aided static and dynamic navigation systems in the placement of zygomatic dental implants. BMC Oral Health.

[6] Pellegrino Gerardo, Lizio Giuseppe, Ferri Agnese, Marchetti Claudio (2021). Flapless and bone-preserving extraction of partially impacted mandibular third molars with dynamic navigation technology. A report of three cases. Int J Comput Dent.

[7] Battista Eric, Gasparro Roberta, Cacciola Maria, Sammartino Gilberto, Marenzi Gaetano (2022). Dynamic navigation system for immediate implant placement in the maxillary aesthetic region. APPS Appl Sci.

[8] Jorba-García A., Figueiredo R., González-Barnadas A., Camps-Font O., Valmaseda-Castellón E. (2019). Accuracy and the role of experience in dynamic computer guided dental implant surgery: an in-Vitro study. Med Oral, Patol Oral Cirugía Bucal.

[9] Sun Ting-Mao, Lee Huey-Er, Lan Ting-Hsun (2020). Comparing accuracy of implant installation with a Navigation System (NS), a Laboratory Guide (LG), NS with LG, and freehand drilling. Int J Environ Res Publ Health.

[10] Pera F., Vocaturo C., Crupi A. (2025). Impact of surgeons' experience on implant placement accuracy using a dynamic navigation system: a cadaver pilot study. Prosthesis.

[11] Pellegrino Gerardo, Lizio Giuseppe, Rossi Fabio (2021). A 4 Mm-Long implant rehabilitation in the posterior maxilla with dynamic navigation technology: a case report after a three-years post-loading Follow-Up. Int J Environ Res Publ Health.

[12] Jung Ronald E., Schneider David, Ganeles Jeffrey (2009). Computer technology applications in surgical implant dentistry: a systematic review. Int J Oral Maxillofac Implants.

[13] Emery Robert W., Merritt Scott A., Lank Kathryn, Gibbs Jason D. (2016). Accuracy of dynamic navigation for dental implant placement-model-based evaluation. J Oral Implantol.

[14] Block Michael S., Emery Robert W. (2016). Static or dynamic navigation for implant placement-choosing the method of guidance. J Oral Maxillofac Surg: Offic J Am Ass Oral Maxillofacial Surg.

[15] Elian Nicolas, Jalbout Ziad N., Classi Anthony J., Wexler Alon, Sarment David, Tarnow Dennis P. (2008). Precision of flapless implant placement using real-time surgical navigation: a case series. Int J Oral Maxillofac Implants.

[16] Wang X.-Y., Liu L., Guan M.-S., Liu Q., Zhao T., Li H.-B. (2022). The accuracy and learning curve of active and passive dynamic navigation-guided dental implant surgery: an in vitro study. J Dent.

